# Safety of biologic and nonbiologic disease-modifying antirheumatic drug therapy in veterans with rheumatoid arthritis and hepatitis B virus infection: a retrospective cohort study

**DOI:** 10.1186/s13075-015-0628-z

**Published:** 2015-05-22

**Authors:** Mary Jane Burton, Jeffrey R Curtis, Shuo Yang, Lang Chen, Jasvinder A Singh, Ted R Mikuls, Kevin L Winthrop, John W Baddley

**Affiliations:** G.V. Sonny Montgomery VA Medical Center, 1500 E Woodrow Wilson Avenue, Jackson, MS 39216 USA; University of Mississippi Medical Center, 2500 North State Street, Jackson, MS 39216 USA; Birmingham VAMC, 700 19th Street S, Birmingham, AL 35233 USA; University of Alabama at Birmingham, 1720 2nd Avenue S, Birmingham, AL 35233 USA; Omaha VAMC; UNMC, 4101 Woolworth Avenue, Omaha, NE 68105 USA; Oregon Health Sciences University, 3181 SW Sam Jackson Park Road, Portland, OR 97239 USA

## Abstract

**Introduction:**

We evaluated the safety of current treatment regimens for patients with RA and HBV in a large US cohort.

**Methods:**

We identified biologic and nonbiologic treatment episodes of RA patients using 1997 to 2011 national data from the US Veterans Health Administration. Eligible episodes had evidence of HBV infection (HBV surface antigen, HBV core antibody, HBV e-antibody and/or HBV DNA) and had a baseline alanine aminotransferase (ALT) <1.5 times the upper limit of laboratory normal within 90 days prior to initiation of a new biologic or nonbiologic DMARD. The main outcome of interest was hepatotoxicity, defined as ALT elevation >100 IU/mL. Results were reported as the cumulative incidence of treatment episodes achieving hepatotoxicity at 3, 6 and 12 months post biologic exposure.

**Results:**

Five hundred sixty-six unique RA patients with HBV contributed 959 treatment episodes. Mean age was 62.1 ± 10.3 years; 91.8% were male. Hepatotoxicity was uncommon, with 26 events identified among 959 episodes (2.7%) within 12 months. Hepatotoxicity was comparable between biologic and nonbiologic DMARDs (2.6% vs. 2.8%, *P* = 0.87). The median time between HBV screening and starting a new RA drug was 504 days (IQR 144, 1,163). Follow-up HBV testing occurred among 14 hepatotoxicity episodes (53.8%) at a median of 202 days (IQR 82, 716) from the date of ALT elevation. A total of 146 (15.2%) treatment episodes received at least one test for HBV DNA at any point in the observation period.

**Conclusions:**

Among US veterans with RA and HBV the risk of hepatotoxicity is low (2.7%), and comparable between biologic and nonbiologic DMARDS (2.8% vs. 2.6%, *P* = 0.87). HBV testing associated with DMARD initiation or hepatotoxicity was infrequent.

**Electronic supplementary material:**

The online version of this article (doi:10.1186/s13075-015-0628-z) contains supplementary material, which is available to authorized users.

## Introduction

Treatment for rheumatoid arthritis (RA) may present unique risks to patients who are also chronically infected with hepatitis B virus (HBV). Agents associated with acute liver injury, such as leflunomide, may be more difficult to tolerate in this setting [1]. Biologic agents and oral glucocorticoids alter host immune responses to HBV infection, which may increase HBV replication with resultant hepatocyte necrosis, a clinical phenomenon known as HBV reactivation [2]. Other agents, such as methotrexate, can cause additive injury over time even in the absence of elevated liver enzyme tests [3].

The sparse literature regarding the safety of current treatments for RA in patients with HBV yields conflicting results. Several case reports document fulminant hepatic failure in RA patients with HBV who were prescribed biologic agents, as well as in patients prescribed traditional disease-modifying antirheumatic drugs (DMARDs) [4-6]. In contrast, small prospective European studies (<100 patients) suggest low to absent risk for hepatotoxicity with biologic DMARDs in patients with rheumatic disease and HBV [7-10]. However, small prospective Asian studies demonstrate increased risk for HBV reactivation with biologic DMARDS and oral glucocorticoids [11,12]. A case-control analysis of the Food and Drug Administration (FDA) Adverse Event Reporting System database found an increased risk of adverse event reporting among patients with RA and HBV who received rituximab, methotrexate and/or oral glucocorticoids and raised concerns regarding increased risk for hepatotoxicity with simultaneous use of multiple DMARDs in these patients [6].

Despite growing concerns regarding appropriate management of viral hepatitis in the setting of RA treatments, the optimal screening for HBV in RA patients is an unsettled issue [13]. Current American College of Rheumatology (ACR) recommendations limit screening to patients with HBV risk factors before prescribing methotrexate or leflunomide [14]. Some experts recommend routine screening prior to the initiation of biologic agents, as well as methotrexate and leflunomide [2]. The FDA recently recommended screening all patients for HBV infection prior use of rituximab [15]. The Centers for Disease Control and Prevention (CDC) advocates routine screening for HBV prior to immunosuppression in all patients [16].

Given the lack of consensus regarding the safety of current treatment regimens for RA in patients with HBV as well as best practices regarding screening for this viral infection, it is important to examine screening practices and outcomes in large registries of RA patients. Herein, we assessed the development of hepatotoxicity and the laboratory evaluation by RA providers to detect this outcome in a large US cohort of veterans with RA and concomitant HBV.

## Methods

### Cohort eligibility criteria

We conducted a retrospective cohort study using electronic health record data from the Veterans Affairs Informatics and Computing Infrastructure [17], linked to administrative data from the Veterans Health Administration Decision Support System from 1997 to 2011. Eligible patients qualified to be in the cohort after they had at least one diagnosis of RA (International Classification of Diseases, Ninth Revision, Clinical Modification (ICD-9-CM) code: 714.X) from a rheumatology provider between October 1, 1997 and December 31, 2011 and a prescription for at least one biologic or nonbiologic DMARD between October 1, 2001 and September 30, 2011. VA prescription data was not available until fiscal year 2002. DMARDs of interest included methotrexate, sulfasalazine, leflunomide, and hydroxychloroquine. Biologics included etanercept, infliximab, adalimumab, rituximab, and abatacept. For comparisons among biologics, we grouped etanercept, infliximab, adalimumab as anti-tumor necrosis factor agents (TNFs). Newer TNFs (for example certolizumab, golimumab) and other biologics (that is tocilizumab) were not used frequently enough to allow for inclusion in this study.

Furthermore, we required laboratory evidence of prior HBV infection, defined as a positive result for any of the following serologic markers prior to the start of follow-up: HBV surface antigen (HBsAg), HBV core antibody (HBcAb), HBV e-antigen (HBeAg), and HBV DNA. At the start of follow-up, termed the ‘index date’, which was anchored at new initiation of a DMARD or biologic, patients must have had a normal or near-normal serum alanine aminotransferase (ALT) level (<1.5 times the upper limit of normal for clinical laboratory monitoring, or 66 IU/ml) measured within the preceding 90 days. National datasets using similar parameters had >95% specificity at excluding American males with significant liver disease [18].

Finally, because the presence of, or treatment for, human immunodeficiency virus (HIV) infection or a hematologic malignancy might affect the risk for hepatotoxicity in patients with concomitant HBV and yield confounding, patients with an ICD-9 code for HIV infection (042.XX) and/or hematologic malignancy (200–208.92) within one year prior to the index date were excluded from the main analysis. However, to maximize generalizability, these patients were included as part of a sensitivity analysis.

### Exposure and outcome assessment

Each drug-specific index date defined a treatment episode. Patients who initiated different therapies could contribute more than one treatment episode. Current exposure was considered as-treated based upon days’ supply (pills or syringes dispensed) or usual dosing intervals (infused biologics). For rituximab, exposure was assumed to extend 12 months after each infusion. Exposure was extended by 90 days after the end of days’ supply or the usual dosing intervals for all other therapies.

The outcome of interest was hepatotoxicity, which was defined as an increase in serum ALT to greater than 100 IU/mL, corresponding to approximately a threefold elevation of population normal levels for males [19,20]. A threefold or greater elevation of ALT has been previously used to identify clinically significant liver enzyme elevation in the setting of viral hepatitis reactivation as well as drug toxicity [21-23]. Suspected cases of hepatotoxicity where confirmed manually by an infectious disease specialist’s review of medical records and assessed for diagnosis of liver failure, hepatic encephalopathy, liver transplant or death within 90 days of the first date of ALT elevation. The cause of death was determined by corresponding death note or diagnosis upon discharge to hospice care.

### Covariates

Covariates were selected based on potential contribution to hepatotoxicity in patients with RA informed by review of the literature [3]. These covariates included demographics, comorbidities (for example diabetes, chronic hepatitis C infection, solid cancer), and other medications used for arthritis (for example oral glucocorticoids). The 12-month period preceding each index date defined the baseline period for assessment of most covariates, except for concomitant medication use, (six months preceding the index date). Comorbid conditions were characterized using ICD-9 codes from provider diagnoses.

### Statistical analysis

Hepatotoxicity was examined in the first one year after treatment initiation. The risk of hepatotoxicity for each drug treatment was reported as a cumulative incidence and quantified as the proportion of patients that met the primary outcome within three, six and twelve months after the index date. Drug-specific comparisons were made using patients receiving sulfasalazine and/or hydroxychloroquine without biologics, methotrexate or leflunomide, which was the referent group. Two-by-two tables were constructed to examine the effect of baseline covariates on hepatotoxicity. Statistical comparisons were made using chi-square or Fisher’s exact test where appropriate. Covariates with central tendency were presented as mean ± standard deviation; those not showing central tendency were measured using interquartile range (IQR).

HBV laboratory screening practices were examined descriptively by plotting the kernel density function of the distribution of time between each treatment episode’s index date and any HBV-related laboratory test. Separate plots were created for laboratory tests before and after the index date, and results were stratified by presence or absence of hepatotoxicity. The purpose of these plots was to descriptively assess the provider’s testing for HBV before initiation of new RA therapies and after treatment when hepatotoxicity was identified. Analyses were performed using SAS 9.2 (SAS Institute, Cary, NC, USA).

### Ethical approval

This study received approval from Institutional Review Boards of the Birmingham VAMC and the G.V. Sonny Montgomery VAMC. Informed consent was not required given the study design.

## Results

### Cohort description

**Figure 1:**
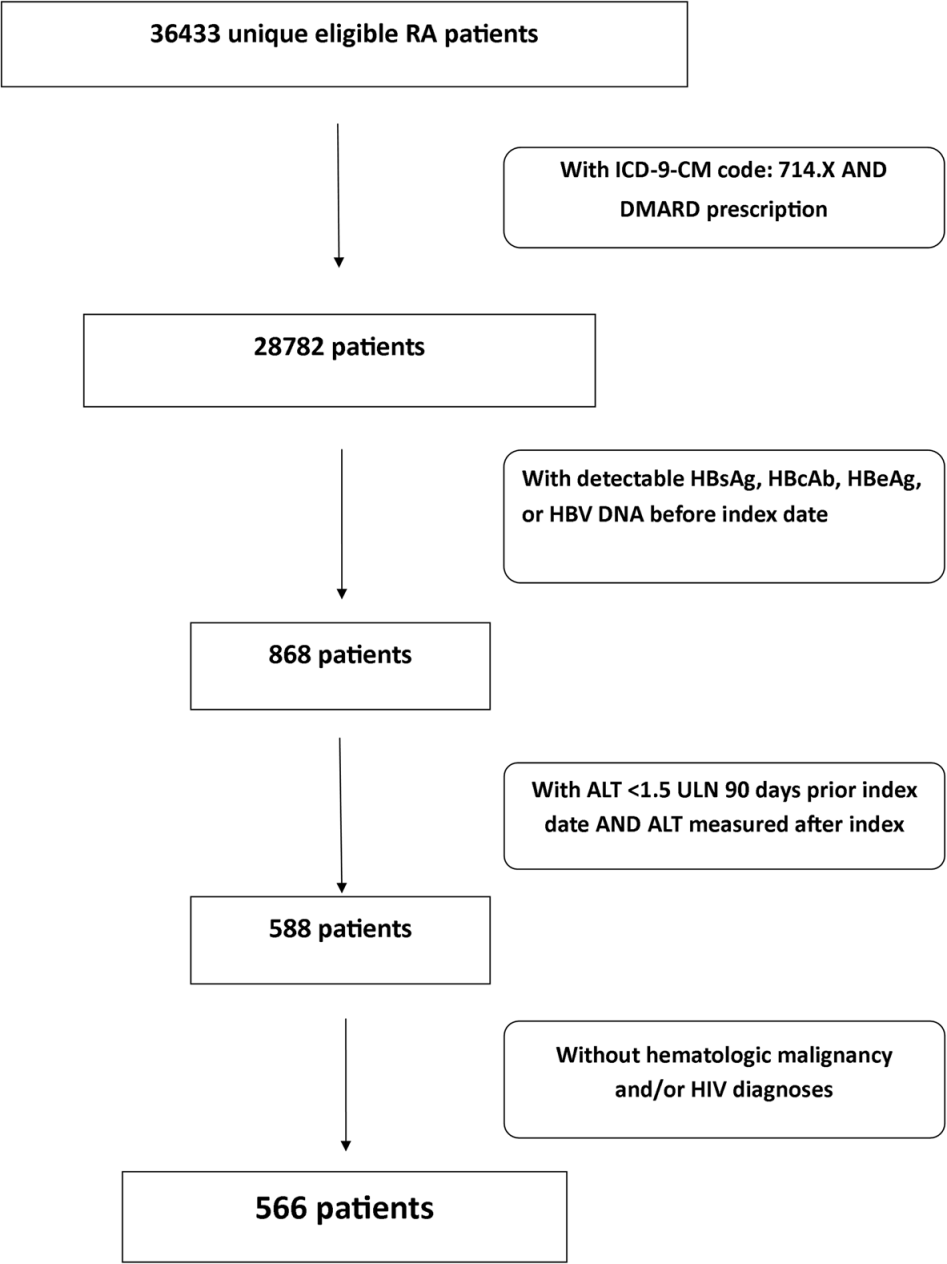
Flow diagram illustrating cohort selection. Index date refers to the date a new DMARD was initiated. DMARD, disease-modifying antirheumatic drugs; HBsAg, hepatitis B surface antigen; HBcAb, hepatitis B core antibody; HBeAg, hepatitis B e-antigen; HBV DNA, hepatitis B DNA.

A cohort of 38,453 patients with RA was identified, from which a total of 566 unique patients contributed 959 treatment episodes (Figure 1; Table 1). Mean age was 62.1 ± 10.3 years; 91.8% of the cohort was male. The most common comorbid diagnoses were hypertension (62.3%) and diabetes mellitus (21.9%); 15.1% were co-infected with hepatitis C.

**Table 1:** **Patient characteristics measured at the start of each medication treatment episode**

**Baseline covariates** **(%)**	**ABA**	**ADA**	**ETA**	**INF**	**LEF**	**MTX**	**RIT**	**SSZ**-**HCQ**	**Total**
	**(** **N** ** = ** **27** **)**	**(** **N** ** = ** **116** **)**	**(** **N** ** = ** **113** **)**	**(** **N** ** = ** **29** **)**	**(** **N** ** = ** **110** **)**	**(** **N** ** = ** **183** **)**	**(** **N** ** = ** **24** **)**	**(** **N** ** = ** **357** **)**	**(** **N** ** = ** **959** **)**
Unique patients	25	113	112	26	98	170	18	302	566
Mean age, years (SD)	60.5 (8.2)	61.0 (9.9)	61.2 (9.7)	57.1 (9.4)	64.5 (11.0)	62.2 (10.6)	62.2 (7.7)	62.3 (10.5)	62.1 (10.3)
Male gender	25 (92.6)	106 (91.4)	108 (95.6)	23 (79.3)	101 (91.8)	167 (91.3)	23 (95.8)	327 (91.6)	880 (91.8)
Medication use six months prior start of treatment episode									
MTX/LEF	18 (66.7)	71 (61.2)	69 (61.1)	20 (69.0)	51 (46.4)	17 (9.3)	12 (50)	145 (40.6)	403 (42.0)
HCQ/SSZ	4 (14.8)	18 (15.5)	19 (16.8)	4 (13.8)	21 (19.1)	68 (37.2)	3 (12.5)	45 (12.6)	182 (19.0)
Oral glucocorticoids	9 (33.3)	43 (37.1)	50 (44.3)	8 (27.6)	39 (35.5)	68 (37.2)	13 (54.2)	117 (32.8)	347 (36.2)
HBV antivirals^1^	1 (3.7)	5 (4.3)	2 (1.8)	0 (0)	0 (0)	3 (1.6)	0 (0)	12 (3.4)	23 (2.4)
Comorbidities^2^									
Hepatitis C	5 (18.5)	18 (15.5)	23 (20.4)	7 (24.1)	6 (5.5)	11 (6.1)	6 (25.0)	69 (19.3)	145 (15.1)
COPD	4 (14.8)	13 (11.2)	19 (16.8)	8 (27.6)	20 (18.2)	5 (19.1)	7 (29.2)	65 (18.2)	171 (17.8)
Hypertension	15 (55.6)	65 (56.0)	65 (57.5)	14 (48.3)	76 (69.1)	112 (61.2)	14 (58.3)	236 (66.1)	597 (62.3)
Diabetes mellitus	4 (14.8)	22 (19.0)	19 (16.8)	2 (6.9)	19 (17.3)	50 (27.3)	4 (16.7)	90 (25.2)	210 (21.9)
Solid cancer	2 (7.4)	6 (5.2)	5 (4.4)	2 (6.9)	17 (15.5)	21 (11.5)	2 (8.3)	31 (8.7)	86 (9.0)

^1^HBV antivirals - lamivudine, entecavir, tenofovir, telbivudine, adefovir, emtricitabine, emtricitabine/tenofovir; ^2^defined by International Classification of Diseases, Ninth Revision (ICD-9) codes and measured 12 months prior to the index date. ABA, abatacept; ADA, adalimumab; ETA, etanercept; INF, infliximab; LEF, leflunomide; MTX, methotrexate; RIT, rituximab; SSZ-HCQ, sulfasalazine/hydroxychloroquine; SD, standard deviation; HBV, hepatitis B virus; COPD, chronic obstructive pulmonary disease.

A total of 650 nonbiologic DMARD treatment episodes and 309 biologic treatment episodes were identified (abatacept 27, rituximab 24, TNFs 258). HBcAb was identified in 827 treatment episodes (86.2%). Detectable HBsAg, HBeAg, and HBV DNA were present in 124 (12.6%), 105 (10.9%) and 28 (2.9%) of all episodes, respectively (episodes could have >1 of these laboratory tests). Over half of the treatment episodes also had detectable HBsAb (531, 55.4%).

The majority (89%) of TNF use was etanercept (113 episodes) and adalimumab (116 episodes). Over half of biologic treatment episodes (58%) were also prescribed concomitant methotrexate or leflunomide within the preceding six months. The prevalence of baseline glucocorticoid use was 36.2%. HBV antiviral prescription occurred either before or within six months of the index date for 23 treatment episodes (2.4%). Of these, the majority of HBV antiviral prescriptions occurred in cases with a detectable HBsAg (21/23, 91.3%); antiviral prescription was associated with a detectable HBsAg (21/124 + HBsAg vs. 2/835 -HBsAg, *P* = 0.001). Lamivudine was the most commonly prescribed HBV antiviral (13/23 episodes, 56.5%). Tenofovir was prescribed in two episodes (8.7%), adefovir and entecavir were each prescribed in four episodes (17.4%).

### Outcome

Of 959 treatment episodes, 26 (2.7%) met the definition of hepatotoxicity (Table 2) occurring among 25 unique patients. Hepatotoxicity was comparable between biologic and nonbiologic DMARDs, with eight episodes identified among the biologics and 18 among traditional DMARDs (2.6% vs. 2.8%, *P* = 0.87). Among biologics, hepatotoxicity occurred in 1/24 rituximab (4.2%), 2/27 abatacept (7.4%) and 5/258 (1.9%) TNF treatment episodes. There was no statistically significant increased risk of hepatotoxicity for any drug-specific comparisons. Based upon medical record review of the 26 cases of hepatotoxicity, there were no cases of liver failure, hepatic encephalopathy or liver transplant. Four deaths were noted, three attributed to infectious causes and one to metastatic bladder cancer.

**Table 2:** **Surveillance for hepatotoxicity during follow**-**up period among treatment episodes**

**Drug**	**Treatment episodes**	**Cumulative events within 12 months** **(%)** ^*^	**Episodes in which any ALT testing occurred during follow** **-** **up period**			**Event rates for hepatotoxicity**		
			**0** **-** **3 month** **(%)** ^**Ϯ**^	**3** **-** **6 month** **(%)** ^**Ϯ**^	**6** **-** **12 month** **(%)** ^**Ϯ**^	**0** **-** **3 month events** **(% )** ^**Ŧ**^	**3** **-** **6 month events** **(%)** ^**Ŧ**^	**6** **-** **12 month events** **(%)** ^**Ŧ**^
Biologic agents	309	8	271	201	179	4	3	1
Abatacept	27	2 (7.4)	24 (88.9)	21 (77.8)	18 (66.7)	0 (0)	1 (4.8)	1 (5.6)
Adalimumab	116	2 (1.7)	103 (88.8)	77 (66.4)	74 (63.8)	1 (1.0)	1 (1.3)	0 (0)
Etanercept	113	3 (2.7)	92 (81.4)	70 (61.9)	65 (57.5)	2 (2.2)	1 (1.4)	0 (0)
Infliximab	29	0 (0)	28 (96.6)	18 (62.1)	14 (48.3)	0 (0)	0 (0)	0 (0)
Rituximab	24	1 (4.2)	24 (100)	15 (62.5)	8 (33.3)	1 (4.2)	0 (0)	0 (0)
Nonbiologic agents	650	18	524	373	317	9	4	5
Leflunomide	110	3 (2.7)	93 (84.5)	71 (64.5)	50 (45.5)	1 (1.1)	1 (1.4)	1 (2.0)
Methotrexate	183	8 (4.4)	163 (89.1)	113 (61.7)	103 (56.3)	3 (1.8)	1 (0.9)	4 (3.9)
Sulfasalazine-Hydroxychloroquine	357	7 (2.2)	268 (75.1)	189 (52.9)	164 (45.9)	5 (1.9)	2 (1.1)	0 (0)
TOTAL (% of total episodes)	959	26 (2.7)	795 (82.9)	574 (59.9)	496 (51.7)	13 (1.6)	7 (1.2)	6 (1.2)

^*^Hepatotoxic events in time period divided by treatment episodes for drug listed in row; ^Ϯ^represents episodes that had at least one ALT test performed in given follow-up time period. Summation of three columns will exceed the total episodes, since an ALT could be performed in all three follow-up periods, unless censuring occurred (failure or end of follow-up period). ^Ŧ^Event rate percentage equals the number of hepatotoxicity events in time period divided by the number of ALT tests performed in same time period for drug row. ALT, alanine aminotransferase.

More hepatotoxic events were detected the first three months after treatment initiation (n = 13) than months 3 to 6 (n = 7) or 6 to 12 (n = 6). Although the majority of hepatotoxic events (n = 20, 77%) were detected in the first six months of therapy, event rates for hepatotoxicity were similar between months 0 to 6 and months 6 to 12 (20/1369, 1.4% vs. 6/496, 1.2%). Based upon the sensitivity analysis that removed the restriction for various diseases, HIV infection (*P* = 0.008), hematologic malignancy (*P* = 0.006), HBV antiviral prescription (*P* = 0.002) and oral glucocorticoid use (*P* = 0.015) were significantly associated with hepatotoxicity (Table 3).

**Table 3:** **Effect of baseline covariates on hepatotoxicity in cohort** (**including HIV infection and hematologic malignancy**, **n** = **1**,**007**)

**Covariate** ^*^	**Covariate present**		**Covariate absent**		***P*** **value** ^Ϯ^
	**Treatment episodes**	**Hepatotoxic episodes** **(%)**	**Treatment episodes**	**Hepatotoxic episodes** **(%)**	
Hepatitis C^§^	158	8(5.1)	849	26(3.1)	0.201
HIV infection^§^	25	4(16.0)	982	30(3.1)	0.008
Hematologic malignancy^§^	23	4(17.4)	984	30(3.1)	0.006
HBV antiviral prescription^¶^	29	5(17.2)	978	29(3.0)	0.002
Oral glucocorticoid prescription^¶^	364	19(5.2)	643	15(2.3)	0.015
Statin prescription^¶^	364	11(3.0)	643	23(3.6)	0.64

^*^Defined within 12 months of index date unless otherwise specified; ^Ϯ^chi-square or Fisher’s exact test; ^§^defined by International Classification of Diseases, Ninth Revision (ICD-9) codes 070.41, 070.44, 070.51, V0262 (hepatitis C), 042.XX (HIV infection), 200–208.92 (hematologic malignancy); ^¶^prescription within six months of index date. HIV, human immunodeficiency virus.

### HBV screening practices

**Figure 2:**
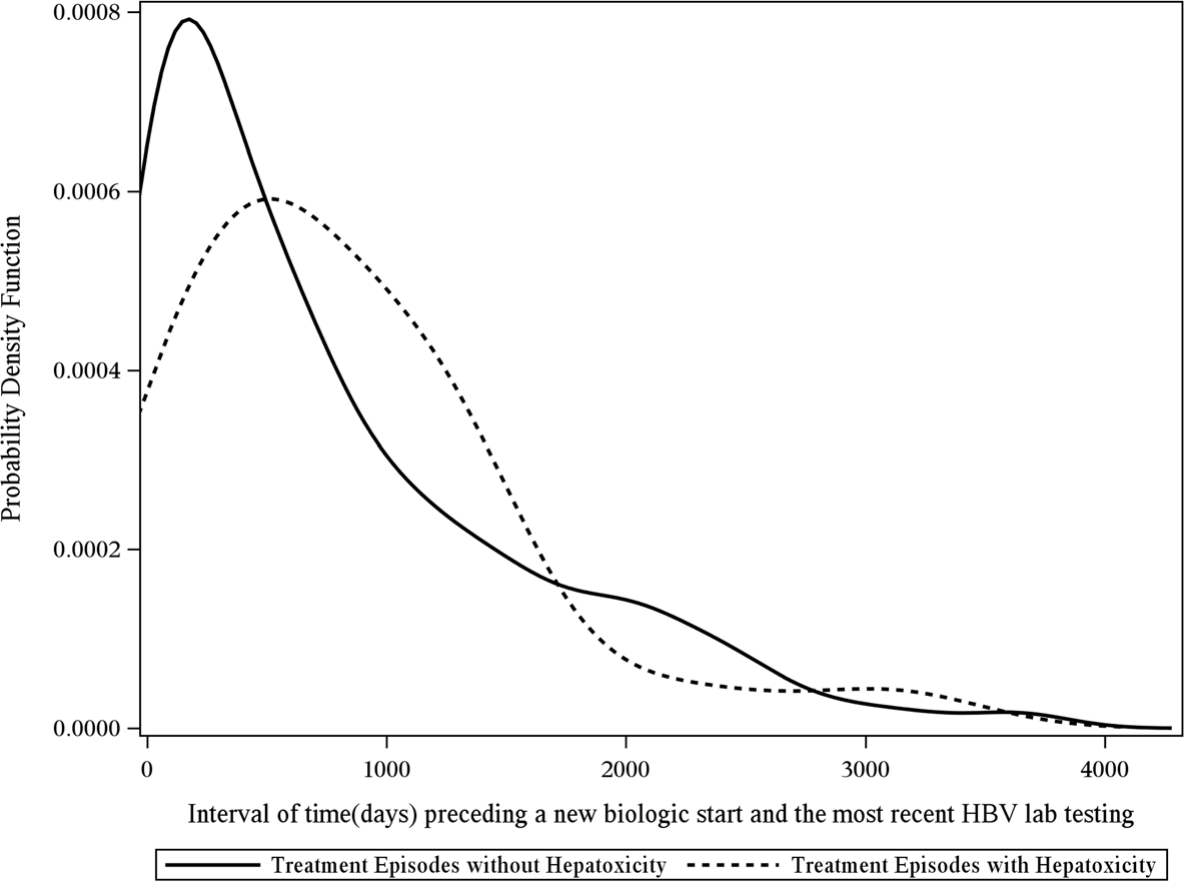
Testing of HBV markers before the index date. Distribution of days (x-axis) between the testing of any HBV laboratory marker (hepatitis B surface antigen, hepatitis B core antibody, hepatitis B e antigen, HBV DNA) prior initiation of a new DMARD (index date), stratified by the presence or absence of hepatotoxicity. (ALT>100 IU/mL). Y axis represents a probability density function that any hepatitis B laboratory marker was ordered at each time point.

**Figure 3:**
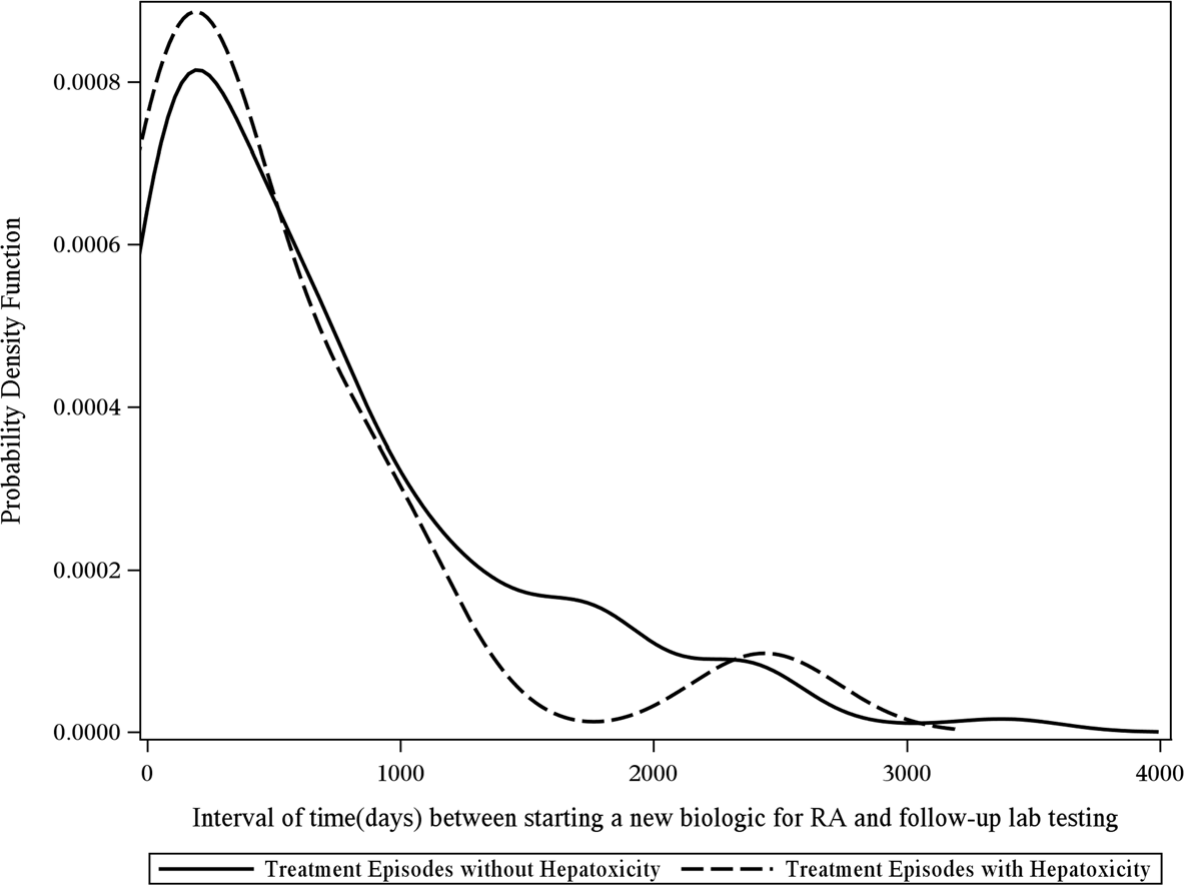
Testing of HBV markers after the index date. Distribution of days (x-axis) between testing of any HBV laboratory marker (hepatitis B surface antigen, hepatitis B core antibody, hepatitis B e antigen, HBV DNA) after initiation of a new DMARD (index date), stratified by the presence or absence of hepatotoxicity. (ALT>100 IU/mL). Y axis represents a probability density function that any hepatitis B laboratory marker was ordered at each time point.

The median time between a preceding test for any HBV laboratory markers and DMARD initiation was 504 days (IQR 144, 1,163) (Figure 2). Following drug initiation, testing for any HBV laboratory markers occurred infrequently, with no observable difference between patients who experienced hepatotoxicity and those who did not (Figure 3). Among 26 episodes of hepatotoxicity, ordering of any HBV laboratory testing occurred in 14 cases (53.8%), at a median of 202 days (IQR 82, 716) from the date of hepatotoxicity to the date the first HBV test was ordered. In patients who did not experience hepatotoxicity, ordering of any HBV laboratory testing occurred in 342 episodes (36.7%), with a median of 494 days (IQR 162, 991) between DMARD initiation and HBV laboratory monitoring. Of the entire cohort, paired testing of HBV DNA occurred prior to DMARD initiation and during follow-up in only 35 instances (3.6%). A total of 146 (15.2%) treatment episodes received at least one test for HBV DNA at any point in the observation period.

## Discussion

This study explored hepatotoxicity in a real-world cohort of RA patients with chronic HBV infection and is among the largest studies to date of RA patients with HBV. Our results show that the overall rate of hepatotoxicity with conventional therapies for RA is low, even for patients with risks for HBV reactivation. In addition, risk of hepatotoxicity was relatively comparable between DMARDs and biologic agents. Among biologics, hepatotoxicity was somewhat more frequent in non-TNFs than TNFs but the difference was not statistically significant. We detected the majority of hepatotoxic events during the initial three months after beginning RA medications. Testing for HBV or HBV reactivation in relation to DMARD or biologic initiation or hepatotoxicity events was infrequent.

Our study is unique in that it examined the safety of RA treatments among the full spectrum of chronic HBV infection, including HBV carriers, resolved HBV infection and isolated HBcAb. HBV carriers retain HBsAg and have increased risk for hepatotoxicity with RA medications when compared to other chronic HBV states [24]. Patients who have HBsAb in addition to HBcAb have ‘resolved’ HBV infection, with little risk for hepatotoxicity from conventional RA treatments. Isolated HBcAb (absence of HBsAb and HBsAg) can signify occult HBV infection, with continued viral replication at low levels in serum or liver tissue [25]. Current ACR guidelines do not address appropriate management of patients with isolated HBcAb [26]. Some experts recommend testing for HBV DNA in this setting to identify higher risk patients who may require more frequent laboratory monitoring and/or antiviral treatment [27].

The observed rate of hepatotoxicity (ALT >100 IU/mL) in our RA cohort of HBV carriers, isolated HBcAb and resolved HBV infection was 1.9% within 12 months of biologic and nonbiologic DMARD initiation, with no serious liver-related sequelae (death, hospitalization, liver transplant) reported as a consequence of treatment for RA. Although the varying cohort compositions, definitions of hepatotoxicity and study duration limit comparison to previously published studies, our low observed rate of hepatotoxicity is reassuring.

In contrast, a retrospective study from Korea reported a 15.9% rate of hepatotoxicity (defined as ALT ≥2 ULN on two consecutive tests) in 88 patients with inflammatory arthritis with isolated HBcAb treated with TNFs ( average follow-up period 24.7 ± 16.4 months after TNF initiation) [28]. A prospective report of Italian rheumatic disease patients found that 34/67 (50.7%) isolated HBcAb-positive patients experienced ALT elevation of ≥2 ULN at a median of 12 months after initiating TNFs in combination with nonbiologic DMARDS (methotrexate, prednisone and/or nonsteroidal anti-inflammatory drugs) [28,29]. No deaths or serious adverse events related to HBV were reported in either study [28,29].

Many studies examining the safety of RA treatments in patients with chronic HBV assess the risk of HBV reactivation, which is characterized by abrupt increases in serum levels of HBV DNA followed by increased transaminases [2]. Although the majority of cases of HBV reactivation in patients with RA are believed to be asymptomatic, severe cases of hepatitis that progress to fulminant hepatic failure and death are reported [30]. A recent systematic review of 122 patients estimated that 12.3% of HBsAg-positive patients receiving TNF and/or DMARD therapy experience HBV reactivation [24]; small prospective studies (<100 patients) estimate HBV reactivation rates of 0 to 5% in HBcAb-positive patients without HBsAg [29]. We could not fully assess for HBV reactivation in our cohort given the infrequent testing of HBV DNA by the treating rheumatologists. Given the low rate of ALT elevation observed, we suspect the rate of clinically significant HBV reactivation was low.

Antiviral prophylaxis is recommended in patients with detectable HBsAg prior to immunosuppression [2]. The evidence for this recommendation stems from studies of HBV patients receiving cytotoxic chemotherapy; however, small studies have suggested a benefit of antiviral prophylaxis in RA patients with detectable HBsAg [31]. In our cohort, treatment episodes prescribed HBV antivirals were more likely to experience hepatotoxicity than those who were not (5/29 (17.2%) vs. 29/978 (3.0%)). We hypothesize that treatment episodes with increased risk for hepatotoxicity were more likely to receive HBV antivirals, as a detectable HBsAg was associated with HBV antiviral prescription.

Among our cohort, screening for HBV in conjunction with DMARD initiation infrequently occurred. The median time between RA treatment initiation and test of any HBV marker (HBsAg, HBcAb, HBeAg, HBV DNA) was 504 days. Testing of any HBV laboratory marker occurred only in roughly one-third of treatment episodes (36.7%) in which hepatotoxicity did not occur. As almost half (55.4%) of treatment episodes demonstrated detectable HBsAb, it is possible providers may have perceived a low risk for hepatotoxicity. However, testing for HBV in conjunction with hepatotoxicity was also infrequent. A follow-up HBV test (HBsAg, HBcAb, and/or HBeAg) was ordered in only just over half of hepatotoxicity episodes (53.8%), with a median time of 202 days from the ALT elevation to performance of any HBV test. This low level of HBV testing likely reflects a limited understanding of appropriate screening and monitoring for HBV infection by providers. In a recent survey of US rheumatologists, 92% of respondents said that they utilized HBsAg for HBV screening but only 54% and 7% ordered HBcAb and HBV DNA, respectively [13]. In this same report, universal screening for HBV prior biologic initiation was affirmed in 69% but dropped to 7% prior any prescription of oral glucocorticoids [13].

Previous publications have suggested increased risks for hepatotoxicity with biologic DMARDS compared to other RA treatments [12,32]; however, we did not detect any significant difference in hepatotoxicity between biologic and nonbiologic DMARDS. As expected, methotrexate and leflunomide had the numerically highest risk for hepatotoxicity. We did find that hematologic malignancy, HIV infection and oral glucocorticoids were associated with the development of hepatotoxicity. Many medications, including those used for cancer and HIV, have strong associations with hepatotoxicity [33-35].

There are limitations to our study. Cohort entry required laboratory detection of an HBV marker, which would not detect episodes where chronic HBV infection was discovered only in the event of hepatotoxicity. Our follow-up period was limited to 12 months after DMARD initiation. HBV reactivation has been reported >2 years of beginning treatment for RA and upon cessation of immunosuppressive medications [33,36]; thus we may have missed later occurring episodes. However, our focus on this early time period was intended to capture the most probable interval in which safety problems could be detected. We used a stringent definition of hepatotoxicity (ALT >100 IU/mL) based upon laboratory criteria, which may overestimate clinical risk with these medications given that no serious medical sequelae ensued. In addition, the infrequent monitoring of HBV DNA did not allow us to distinguish ALT elevations as HBV reactivation. We could only review the records of care within the VHA health care system. We cannot exclude the possibility that some patients may have had monitoring performed, hepatotoxicity detected, and received treatment for HBV by outside providers. Lastly, our cohort was largely comprised of older US males with RA and may not be generalizable to other RA populations.

## Conclusions

We report a low rate of hepatotoxicity among a large cohort of US veterans with RA and HBV infection who were prescribed conventional RA therapies. We found comparable rates of hepatotoxicity between biologic and nonbiologic DMARDS. Screening for HBV in conjunction with DMARD initiation and/or at the time of identified hepatotoxicity, was infrequent.

### Ethics approval

This study was conducted with the approval of the Institutional Review Boards at the Jackson VAMC and the Birmingham VAMC.

## Abbreviations

ALT: alanine aminotransferase
DMARD: disease-modifying antirheumatic drug
HBcAb: hepatitis B core antibody
HBeAg: hepatitis B e-antigen
HBsAg: hepatitis B surface antigen
HBV: hepatitis B virus
HIV: human immunodeficiency virus
ICD-9-CM: International Classification of Diseases, Ninth Revision, Clinical Modification
IQR: interquartile range
RA: rheumatoid arthritis
TNF: anti-tumor necrosis factor agent.

## References

[1] Aithal GP (2011). Hepatotoxicity related to antirheumatic drugs. Nat Review Rheumatol..

[2] Calabrese LH, Zein NN, Vassilopoulos D (2006). Hepatitis B virus (HBV) reactivation with immunosuppressive therapy in rheumatic diseases: assessment and preventive strategies. Ann Rheum Dis..

[3] Visser K, van der Heijde DM (2009). Risk and management of liver toxicity during methotrexate treatment in rheumatoid and psoriatic arthritis: a systematic review of the literature. Clin Exp Rheum..

[4] Bae J, Sohn J, Lee H, Park HS, Hyun YS, Kim TY (2012). A fatal case of hepatitis B virus (HBV) reactivation during long-term, very-low-dose steroid treatment in an inactive HBV carrier. Clin Mol Hepatol..

[5] Ito S, Nakazono K, Murasawa A, Mita Y, Hata K, Saito N (2001). Development of fulminant hepatitis B (precore variant mutant type) after the discontinuation of low-dose methotrexate therapy in a rheumatoid arthritis patient. Arthritis Rheum..

[6] Oshima Y, Tsukamoto H, Tojo A (2013). Association of hepatitis B with antirheumatic drugs: a case-control study. Mod Rheumatol..

[7] Vassilopoulos D, Apostolopoulou A, Hadziyannis E, Papatheodoridis GV, Manolakopoulos S, Koskinas J (2010). Long-term safety of anti-TNF treatment in patients with rheumatic diseases and chronic or resolved hepatitis B virus infection. Ann Rheum Dis..

[8] Charpin C, Guis S, Colson P, Borentain P, Mattéi JP, Alcaraz P (2009). Safety of TNF-blocking agents in rheumatic patients with serology suggesting past hepatitis B state: results from a cohort of 21 patients. Arthritis Res Ther..

[9] Mitroulis I, Hatzara C, Kandili A, Hadziyannis E, Vassilopoulos D (2013). Long-term safety of rituximab in patients with rheumatic diseases and chronic or resolved hepatitis B virus infection. Ann Rheum Dis..

[10] Biondo MI, Germano V, Pietrosanti M, Canzoni M, Marignani M, Stroffolini T (2014). Lack of hepatitis B virus reactivation after anti-tumor necrosis factor alpha agents therapy in antibody to hepatitis B core antigen positive/hepatitis B surface antigen negative subjects with chronic inflammatory arthropathies. Eur J Intern Med..

[11] Tan J, Zhou J, Zhao P, Wei J (2012). Prospective study of HBV reactivation risk in rheumatoid arthritis patients who received conventional disease-modifying antirheumatic drugs. Clin Rheumatol..

[12] Urata Y, Uesato R, Tanaka D, Kowatari K, Nitobe T, Nakamura Y (2011). Prevalence of reactivation of hepatitis B virus replication in rheumatoid arthritis patients. Mod Rheumatol..

[13] Stine JG, Khokhar OS, Charalambopoulus J, Shanmugam VK, Lewis JH (2010). Rheumatologists' awareness of and screening practices for hepatitis B infection prior initiating immunomodulatory therapy. Arth Care Res (Hoboken)..

[14] Saag KG, Teng GG, Patkar N, Anuntiyo J, Finney C, Curtis JR (2008). American College of Rheumatology 2008 recommendations for the use of nonbiologic and biologic disease-modifying antirheumatic drugs in rheumatoid arthritis. Arthritis Rheum..

[15] Food and Drug Administration Drug Safety Communication. Boxed warning and new recommendations to decrease risk of hepatitis B reactivation with the immune-suppressing and anti-cancer drugs Arzerra (ofatumumab) and Rituxan (rituximab). http://www.fda.gov/Drugs/DrugSafety/ucm366406.htm. Accessed September 25, 2013.

[16] Weinbaum CM, Williams I, Mast EE, Wang SA, Finelli L (2008). Recommendations for identification and public health management of persons with chronic HBV infection. MMWR Recomm Rep..

[17] VIReC Research User Guides Index. http://vaww.virec.research.va.gov/RUGs/RUGs-Index.htm. Accessed May 8, 2013.

[18] Ruhl CE, Everhart JE (2012). Upper limits of normal for alanine aminotransferase activity in the United States population. Hepatology..

[19] Piton A, Poynard T, Imbert-Bismut F, Khalil L, Delattre J, Pelissier E (1998). Factors associated with serum alanine transaminase activity in healthy subjects: consequences for the definition of normal values, for selection of blood donors, and for patients with chronic hepatitis C. Hepatology..

[20] Food and Drug Administration. Drug-induced liver injury: premarketing clinical evaluation. https://www.fda.gov/Drugs/Guidances/ucm064993.htm. Accessed February 7, 2014.

[21] Temple R (2006). Hy's law: predicting serious hepatotoxicity. Pharmacoepidemiol Drug Safe..

[22] Lok AS, Lai CL, Wu PC, Leung EK, Lam TS (1987). Spontaneous hepatitis B e antigen to antibody seroconversion and reversion in Chinese patients with chronic hepatitis B virus infection. Gastroenterology..

[23] Visser K, Katchamart W, Loza E, Martinez-Lopez JA, Salliot C, Trudeau J (2009). Multinational evidence-based recommendations for the use of methotrexate in rheumatic disorders with a focus on rheumatoid arthritis: integrating systematic literature research and expert opinion of a broad international panel of rheumatologists in the 3E Initiative. Ann Rheum Dis..

[24] Lee YH, Bae SC, Song GG (2013). Hepatitis B virus reactivation in HBsAg-positive patients with rheumatic diseases undergoing anti-tumor necrosis factor therapy or DMARDs. Int J Rheum Dis..

[25] Lok ASF, McMahon BJ (2009). Chronic hepatitis B: update 2009. Hepatology..

[26] Singh JA, Furst DE, Bharat A, Curtis JR, Kavanaugh AF, Kremer JM (2012). 2012 update of the 2008 American College of Rheumatology recommendations for the use of disease-modifying antirheumatic drugs and biologic agents in the treatment of rheumatoid arthritis. Arthritis Care Res..

[27] Winthrop KL, Calabrese LH (2011). Let the fog be lifted: screening for hepatitis B virus before biological therapy. Ann Rheum Dis..

[28] Kim YJ, Bae SC, Sung YK, Kim TH, Jun JB, Yoo DH (2010). Possible reactivation of potential hepatitis B virus occult infection by tumor necrosis factor-alpha blocker in the treatment of rheumatic diseases. J Rheumatol..

[29] Caporali R, Bobbio-Pallavicini F, Atzeni F, Sakellariou G, Caprioli M, Montecucco C (2010). Safety of tumor necrosis factor α blockers in hepatitis B virus occult carriers (hepatitis B surface antigen negative/anti-hepatitis B core antigen positive) with rheumatic diseases. Arthritis Care Res (Hoboken)..

[30] Droz N, Gilardin L, Cacoub P, Berenbaum F, Wendling D, Godeau B (2013). Kinetic profiles and management of hepatitis B virus reactivation in patients with immune-mediated inflammatory diseases. Arthritis Care Res (Hoboken)..

[31] Lan JL, Chen YM, Hsieh TY, Chen YH, Hsieh CW, Chen DY (2011). Kinetics of viral loads and risk of hepatitis B reactivation in hepatitis B core antibody-positive rheumatoid arthritis patients undergoing anti-tumour necrosis factor alpha therapy. Ann Rheum Dis..

[32] Carroll MB, Forgione M (2010). Use of tumor necrosis factor alpha inhibitors in hepatitis b surface antigen-positive patients: a literature review and potential mechanisms of action. Clin Rheum..

[33] Hoofnagle JH (2009). Reactivation of hepatitis B. Hepatology..

[34] den Brinker M, Wit FW, Wertheim-van Dillen PM, Jurriaans S, Weel J, van Leeuwen R (2000). Hepatitis B and C virus co-infection and the risk for hepatotoxicity of highly active antiretroviral therapy in HIV-1 infection. AIDS..

[35] Xuan D, Yu Y, Shao L, Wang J, Zhang W, Zou H (2014). Hepatitis reactivation in patients with rheumatic diseases after immunosuppressive therapy-a report of long-term follow-up of serial cases and literature review. Clin Rheumatol..

[36] Kim PS, Ho GY, Prete PE, Furst DE (2012). Safety and efficacy of abatacept in eight rheumatoid arthritis patients with chronic hepatitis B. Arthritis Care Res (Hoboken)..

